# Spatial correlation between electricity generation and economic scale in Africa

**DOI:** 10.1371/journal.pone.0300627

**Published:** 2024-06-24

**Authors:** Huijuan Fu, Guoqing Lyu, XiuQing Liu, Haining Jiang

**Affiliations:** 1 College of Mathematics and Computer Science, Zhejiang Normal University, Jinhua, China; 2 School of Resources, Environment and Architectural Engineering, Chifeng University, Chifeng, China; 3 College of Geography and Environmental Sciences, Zhejiang Normal University, Jinhua, China; Sichuan Agricultural University, CHINA

## Abstract

This study attempts to determine whether there is a spatial correlation between electricity generation and economic scale promoting coordinated development in Africa. We explore the spatial similarity and gray correlation degree between electricity generation and economic scale in Africa since the 21st century by adopting barycenter coupling and Gray Correlation Analysis method. We argue that there is a strong correlation between electricity generation and economic scale. Our findings indicate a significant spatial difference in electricity generation, mainly concentrated in Northern and Southern Africa. Furthermore, spatial pattern remains largely consistent over time, mirroring trends observed at the economic scale. Electricity generation and economic scale were concentrated in six countries- South Africa, Egypt, Algeria, Nigeria, Morocco, and Libya- and did not change significantly over time. A correlation analysis between electricity generation and the economic scale further confirmed this, with a linear coefficient of 0.907. Both the gravity centers of economic scale and electricity generation in Africa move farther in the north-south direction than in the East-West direction, with the former showing a Southwest-Northeast-Southwest track feature and the latter a Northeast-Southwest track feature. The spatial distribution of the gravity centers of electricity generation and the gravity centers of the economic scale in Africa are highly consistent; electricity generation highly correlates with the economic scale, consistent with the research conclusion obtained by the Gray Correlation Analysis method. This study suggests the coordinated development of electricity generation and economic scales in various African countries.

## Introduction

The rapid development of the world economy, population growth, and lifestyle changes has promoted a rapid increase in the demand for electricity. Electric power is an indispensable component of the economic scale, and the "barometer" and "thermometer" of economic operation. As an important measure of electricity capacity, an increase in electricity generation signals a better quality of life and greater wealth creation [1], whereas a lack of generating capacity may have a significant negative impact on a country’s economic scale [2, 3]. According to the World Energy Statistical Yearbook 2020, the world’s total electricity generation in 2019 was 27004.7 TWh, with a quarter-on-quarter growth (QOQ) of 1.32% [4]. In the same year, the GDP reached 87.56 trillion US dollars, with a QOQ growth rate equal to that of electricity generation (1.52%), displaying a good trend of coordinated development between electricity generation and economic scale. By 2019, the global electricity connection rate had increased to 90%, and the number of people without electricity had decreased to 759 million, in which fossil fuel power generation played an important role. Increased greenhouse gas emissions also lead to environmental pollution. Over time, an increasing number of countries will pursue sustainable development as their general goal; thus, developing renewable energy is the main option, as it can reduce carbon dioxide emissions and stimulate economic prosperity. Hydroenergyr has proven to be the most effective in promoting economic development, followed by wind and solar energy [5]. Hydropower dams are among the main sources of electricity and the largest renewable source of power generation worldwide. Despite their efficiency and environmental benefits, hydropower dams have considerable negative impacts on ecosystem due to factors such as design, location, and efficiency [6, 7]. Therefore, evaluating the economic–social performance of hydropower projects has also received attention [8, 9].

Many studies have confirmed that the electricity supply is still one of the important obstacles to future economic growth, which has attracted increasing attention from scholars at home and abroad. In particular, the existing research on the relationship between electricity and economic scale mainly focuses on the causal relationship between electricity consumption (or supply) and economic scale. The results are mainly based on an economic perspective [10, 11] and the national scale via the cointegration test method [12] and the causality test model [13, 14]. For example, Morimoto [15] tested the relationship between electricity supply and economic scale in Sri Lanka and established bidirectional causality between electricity supply and real GDP. Ateba et al. [16] further refined the research content and showed that electricity supply in South Africa had an unidirectional effect on an industrial scale through a quantitative research design. The negative impact of unsustainable electricity supply on industrial development is also evaluated. Balcilar M et al. [17] studied the interaction between electricity consumption, real GDP, and CO_2_ emissions in Pakistan and found a one-way causal relationship between economic growth and electricity consumption. In contrast, there are few studies on the relationship between electricity generation and economic scale, but the conclusion has confirmed a high correlation between them. For example, relevant studies by the Institute of Water Resources and Electric Power Information of the Ministry of Energy of China indicate a high correlation between electricity and economic scale growth, and the power elasticity of developing countries is generally greater than that of developed countries [18]. Ma Zhizhong [19] analyzed electricity generation growth in the United States, the former Soviet Union, Japan, and other countries as their per capita GDP increased from $200 to $10,000. This analysis serves as a basis for predicting China’s future electricity development.

By 2019, 80 percent of the 600 million people in sub-Saharan Africa live in rural areas and had no access to electricity [20]. For those with access, electric services are the most expensive and least reliable world [21]. As the continent with the concentration of developing countries, sub-Saharan Africa still comprises nearly 30 nations. The average electricity coverage rate remains below 60%, with an electricity connection rate of only 46.8%. As many as 570 million people, which constitutes three-quarters of the current global population, lack access to electricity [22]. Electricity shortages are estimated to cost the African continent approximately2%-4% of GDP per year [23], and the continent needs approximately$55 billion in investment per year by 2030 to solve its energy challenges [24]. However, this has not attracted significant attention from scholars worldwide. McKinsey [25] found that promoting the development of the electricity or energy sector could promote the expansion of the African economic scale. Ebhota [26] listed the lack of electricity generation as one of the main constraints on the growth of such a scale. This is consistent with Shokoya [27] and Trotter et al. [28], who argued that electricity shortages are essential obstacles to the growth of the African economy. Hlalefangr [29] used South Africa as an example to verify the causal relationship between renewable electricity generation and economic scale, revealing the unidirectional promoting effect of the former on the latter. Lawal [30] investigated sub-Saharan African economies and used adynamic panel threshold regression model to explore the relationship between their economic scale and electricity generation, confirming obvious differences in the causal relationship in different times and regions.

In general, studies on the relationship between global electricity generation and economic scale mainly focus on developed countries and a few typical developing countries and explore the causal mechanism mainly from the economics perspective. In contrast, most studies on the relationship between electricity generation and economic scale for atypical region or country in Africa are mainly based on an analysis from an economic perspective. However, there is a lack of research from the perspective of visual expressions in geographic spaces. Africa is generally ignored when depicting the coupling between electricity and economic-scale space and its interaction mechanism. As an underdeveloped region, Africa’s economic scale has grown rapidly since the 21st century. Therefore, what is the role of electricity generation on an economic scale? What about the spatial correlation between electricity generation and economic scale in different countries? These are urgent questions that must be addressed. Based on this, this study adopts the spatial interpolation method in Arc GIS and the gray correlation analysis method in DPS to analyze the spatial pattern of electricity generation and economic scale in Africa. Then, the spatial correlation of electricity generation on am economic scale is analyzed. This study aims to offer a theoretical reference and decision-making basis for the long-term planning of the economic scale of the African continent. It seeks to inform the formulation of a sustainable development strategy for energy and facilitate the coordinated development of electricity generation and economic scale. Ultimately, the goal is to promote African countries’ economic development and social progress and achieve sustainable energy development.

The remainder of this paper is organized as follows. Section 2 briefly describes the correlation mechanisms between electricity generation and economic scale. Section 3 briefly introduces the methodology and data sources. Sections 4, 5, and 6 present an empirical analysis and an explanation of the results, respectively. Finally, conclusions and implications for further research are discussed in Section 7.

### The correlation mechanisms between electricity generation and economic scale

Electric power plays a vital role in the development of the national economy, providing energy supply and electricity support for the development of various industries. There is a close relationship between electricity generation and economic scale.

A stable electricity supply is the basis for economic development. Electricity is an important secondary energy source that can be directly consumed [31]. The amount of electricity generated affects energy availability, thereby driving changes in human capital, which can have lasting effects on socio-economic development [32]. If less electricity is generated, prices will rise accordingly; consequently, people may find it unaffordable, leading to a reduction in their quality of life, which can in turn impact human development and economic growth [33]. Conversely, electricity generation and industrial growth have a long-term stable relationship [34]. Electricity generation is a crucial factor in industrial output, especially for pig iron, crude steel, aluminum, and other major industrial products. The use of electricity, especially renewable electricity, compared with non-renewable energy sources such as coal and oil, can reduce production costs. Furthermore, it promotes sustainable economic growth while increasing profits [35].

## Methods and data

### Research methods

#### Barycenter coupling analysis

Originating in physical science, the concept of coupling refers to the dynamic relationship between systems that mutually affect each other [36, 37]. Coupling refers to the phenomenon in which two or more systems or motion modes affect each other through various interactions. The degree of coupling can effectively measure the degree of coordination between systems [37]. This study builds upon Professor Fan Jie’s research method [38], which investigates the spatial coupling of China’s economic and population gravity centers. Here, we calculate the spatial overlap and variation consistency between electricity generation and economic-scale gravity centers in Africa. Additionally, we examine the static and dynamic conditions of the coupling between electricity generation and economic scale-gravity centers in Africa. Our objective is to explore the spatial correlation between electricity generation and economic scale in Africa by analyzing the spatial coupling relationship of gravity centers.

Spatial overlap is expressed by the spatial distance between the gravity centers of electricity generation and the gravity centers of economic scale in Africa. A shorter spatial distance indicates a higher spatial overlap, while a longer distance indicates a lower one. In the formula, E and P represent the coordinates of the electricity generation gravity center and the economic-scale gravity center of different gravity centers in the same year, which are (*x*_*E*_, *y*_*E*_) and (*x*_*p*_, *y*_*p*_) respectively. The formula is as follows:

$$S=d_{G_{E}G_{P}}=\sqrt{{\left(x_{E}-x_{p}\right)}^{2}+{\left(y_{E}-y_{p}\right)}^{2}}$$ (1)

The consistency of change in the formula is reflected by the vector intersection Angle *θ* of the displacement between the gravity centers of the electricity generation and the gravity centers of economic scale in Africa relative to the time point. A smaller *θ* indicates a more consistent change. The value range of *θ* is 0°≤θ≤180°, and the change consistency index can be represented by its cosine value C (-1≤C≤1). The larger the value is, the more consistent the change. C = 1 means completely in the same direction; when C = -1, it means completely in the opposite direction. Δx and Δy represent the change in longitude and latitude of the gravity center from the previous time point. According to the law of cosine:

$$\begin{array}{l} C=\cos\theta \\ =\frac{\left(\Delta x_{E}^{2}+\Delta y_{E}^{2}\right)+\left(\Delta x_{p}^{2}+\Delta y_{p}^{2}\right)-\left[{\left(\Delta x_{E}-\Delta y_{E}\right)}^{2}+{\left(\Delta x_{p}-\Delta y_{p}\right)}^{2}\right]}{2\sqrt{\left(\Delta x_{E}^{2}+\Delta y_{E}^{2}\right)\left(\Delta x_{p}^{2}+\Delta y_{p}^{2}\right)}}\\ =\frac{\Delta x_{E}\Delta x_{p}+\Delta y_{E}\Delta y_{p}}{\sqrt{\left(\Delta x_{E}^{2}+\Delta y_{E}^{2}\right)\left(\Delta x_{p}^{2}+\Delta y_{p}^{2}\right)}} \end{array}$$ (2)

#### Gray correlation analysis

Gray correlation analysis theory was proposed by Professor Deng Julong in 1982 [39]. It is mainly used to analyze the degree of similarity or dissimilarity between the development trends of factors in the gray system, known as the "gray correlation degree.” This method is essential for measuring the closeness of the correlation among factors [39]. Compared with mathematical statistical methods, gray correlation analysis can fully consider the relationships between factors and has no special requirements on the amount or relationships of the data. The greater the gray correlation degree, the stronger the influence of the influencing factor index on the economic scale, and vice versa. The calculation involves five steps: first, identify the reference data column and the comparison data column; second, perform non-dimensional processing of index data; third, measure the absolute difference between the corresponding elements of the comparative data column and the reference data column one by one; fourth, calculate the correlation coefficient; and finally, calculate the gray correlation degree.

### Data sources

Since the beginning of the 21st century, Africa has experienced rapid growth in economic scale and electricity generation, and the causal relationship is significant. We selected the period from 2000 to -2019 and obtained data on electricity generation and economic scale (expressed as “GDP”) from the African Statistical Yearbook 2001–2020 to ensure data consistency throughout the study. We excluded Western Sahara and certain islands for data continuity and accessibility. Moreover, South Sudan declared independence on July 9, 2011. To maintain data consistency, Sudan and South Sudan were combined, totaling 53 countries included in the study. The study compiled yearly statistics from these 53 countries over 20 years, covering the years 2000 to 2019. In cases where data for individual indicators or countries were missing, we used data from adjacent years as substitutes, or we summed and averaged data from previous and subsequent years.

## Spatial pattern of electricity generation and economic scale

### Spatial pattern of electricity generation

**Fig 1:**
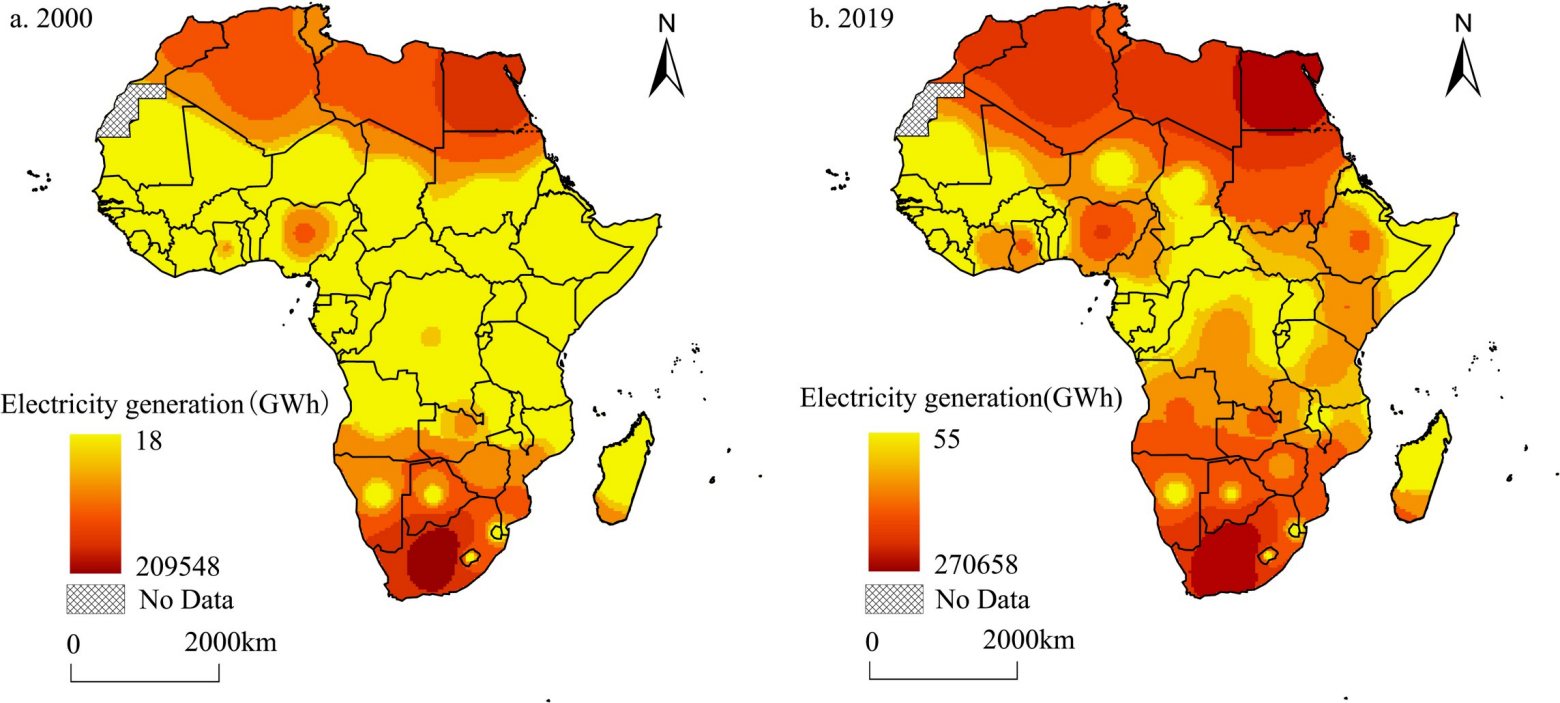
Spatial distribution change of electricity generation in Africa (2000–2019). The spatial distribution pattern of electricity generation in Africa in 2000 and 2019 is on the left and right, respectively. The closer the color is to red, the greater the electricity generation. The closer the color is to yellow, the less electricity is generated.

The spatial patterns of electricity generation in Africa in 2000 and 2019 were analyzed using the inverse distance weight interpolation method in ArcGIS. The spatial variation of electricity generation in Africa is significant, mainly concentrated in Northern and Southern Africa, exhibiting a “double core” spatial structure of concentration centered on South Africa and Egypt. Spatial patterns did not change significantly over time (Fig 1). In 2000, the total electricity generation in Africa was 438.9 TWh, accounting for only 2.9% of the world’s total electricity generation, with South Africa and Egypt accounting for 48.5% and 16.2% respectively, or 64.70% in total. In comparison, electricity generation in West, Central, and East Africa is generally lower, and these regions are the main concentrated areas on the continent that have not yet been electrified. Second, electricity generation in Algeria (North Africa) and Nigeria (West Africa) was also higher, accounting for 5.9% and 3.4% of Africa’s total electricity generation, respectively. In 2019, Africa’s total electricity generation was 875.3 TWh, accounting for 3.2% of the world’s total electricity generation, with a growth rate of 101.5% compared with 2000. Its electricity generation capacity is still concentrated in Northern and Southern Africa, with South Africa and Egypt being the highest, accounting for 31.1% and 23.0% of Africa’s total electricity generation capacity, respectively, for a total of 54.1%. This is followed by Algeria, Libya, Morocco, and Nigeria, whose electricity generation capacities grew relatively rapidly (9.2%, 4.5%, 4.0%, and 3.7%, respectively). These figures indicate that the spatial pattern of the relative concentration of electricity generation in North and South Africa did not change significantly over time and that the agglomeration and scale effects were more prominent. As the electricity generation in West, Central, and East Africa increase significantly, the spatial differences between the regions gradually narrowed.

It is evident that countries such as South Africa, Egypt, and Algeria consistently stand out as the highest electricity generation areas on the African continent. South Africa’s electricity industry has a long history and operates at a high level, and it is the only country in Africa with nuclear electricity plants. Over 95% of South Africa’s electricity is provided by the national electricity company Eskom [40], which boasts the world’s largest dry-cooled electricity plant and ranks the seventh-largest producer of electricity. Furthermore, Sasol’s status as the sole international company producing synthetic fuels from coal liquefaction on a large scale, coupled with its mature technology ranking among the world’s best, has positioned South Africa as one of the foremost electricity producers in Africa. In contrast, Egypt is rich in natural gas, which accounts for 76.4% of the total fuel consumed for electricity, and has developed various renewable energy source, that account for 9% of the country’s total electricity generation. Given its relative abundance of electricity, Egypt has formed an electricity agglomeration center in northern Africa with neighboring countries such as Libya and Algeria [41]. Algeria is the largest natural gas producer in Africa, the second largest gas supplier in Europe, and one of Africa’s top three oil producers, with gas and oil accounting for 64.8% and 34.6% of the country’s electricity generation, respectively. Additionally, a series of solar and wind renewable energy projects have been planned for the Saharan Desert [42], and the country has long been among the top electricity producers in Africa. Nigeria is Africa’s first oil-producing and exporting country in Africa, and its proven gas and oil reserves are the first and second, respectively. Its electricity generation capacity is also high, but there is a gap compared to the three countries above, especially the low per capita electricity generation capacity. Only 40% of Nigeria’s population is connected to the national electricity grid, and this connected population faces power problems 60% of the time [43, 44]. Such capacity seriously restricts the economic scale of the country. It is related to the relatively backward infrastructure, insufficient government investment in electricity plants, and the inability of the power grid to cover all areas of Nigeria [45]; other factors are also closely related. Nevertheless, electricity shortages remain a common problem in by many African countries [46], and the problems of insufficient electricity generation infrastructure and equipment are prominent. Many small-scale and oil-based electricity generation systems make generation costly, making enhancing and meeting the electricity demand challenging. Coupled with the poor management of African governments, electricity development in Africa has a long way to go.

### Spatial pattern of economic scale

**Fig 2:**
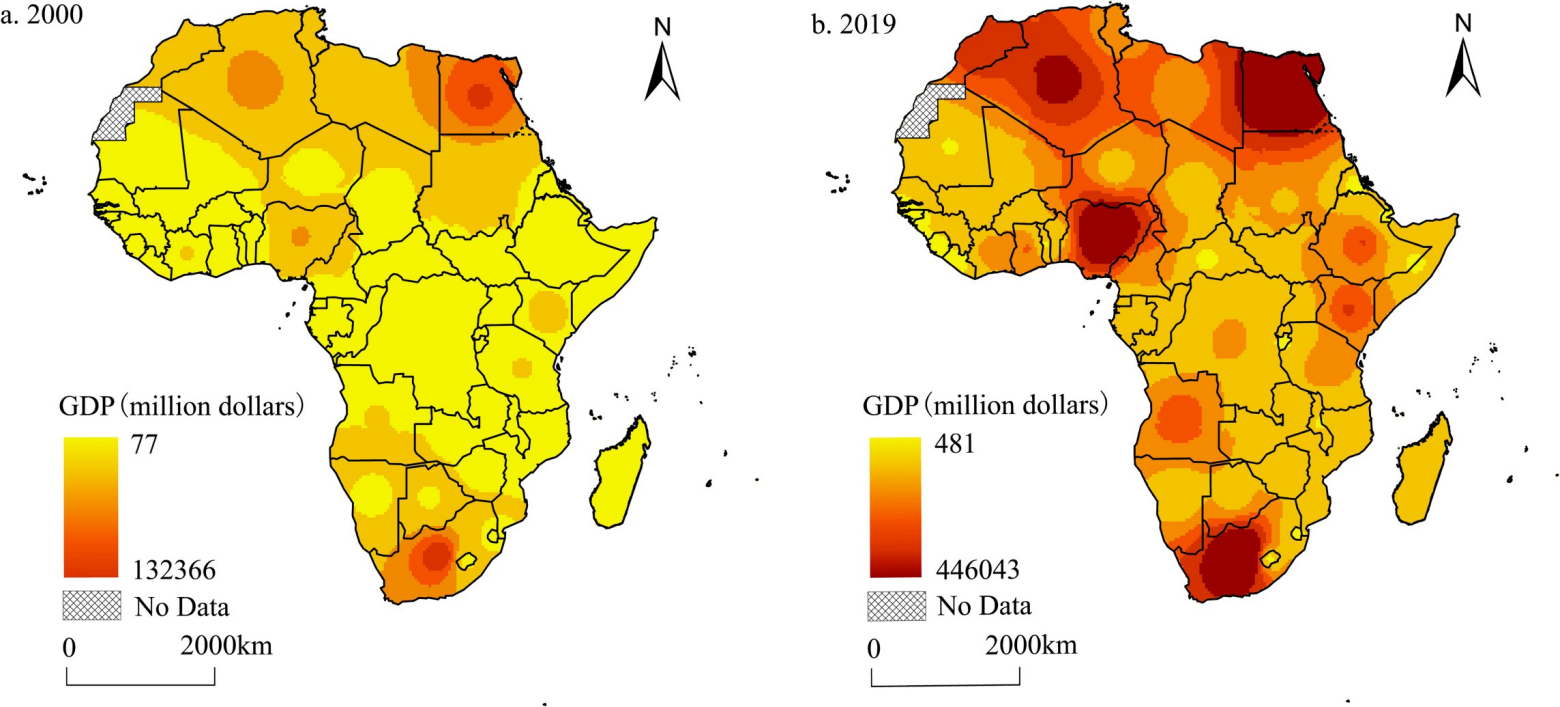
Spatial distribution change of economic scale in Africa (2000–2019). The spatial distribution patterns of Africa’s economic scale in 2000 and 2019 are shown on the left and right sides, respectively. The closer the color is to red, the larger the economy. The color is about yellow, indicating a smaller economy.

The inverse distance weight interpolation method in ArcGIS was used to analyze the spatial pattern of the African economic scale in 2000 and 2019. The results indicate that the overall spatial pattern is more similar to that of African electricity generation and does not change significantly over time. High-value areas were mainly concentrated in Northern and Southern Africa, and the results showed a gradual upward trend. In particular, the original economic scale high-value areas have a more significant clustering trend, which in turn highlights the significant role of the clustering effect in the growth of the African economy (Fig 2). Specifically, in 2000, Africa’s total GDP was $0.59 trillion, with a relatively small economy and an uneven spatial pattern, exhibiting a "dual-core" agglomeration pattern centered on South Africa in Southern Africa and Egypt in Northern Africa. Their GDP accounts for 22.5% and 16.6% of Africa’s total GDP, respectively, for a total of 40%. In 2019, Africa’s total GDP reached $2.43 trillion, up 312.2% from 2000, but its share the of total global GDP remained low at 2.8%. Compared with 2000, the economic scale of African countries in 2019 generally saw a significant increase. In addition to South Africa, Egypt and other countries with originally high GDP growth also exhibited a relatively rapid increase in their economic scale. Algeria, Nigeria, Morocco, and other countries have also grown rapidly. Nigeria has replaced South Africa as the largest economy in Africa, and the GDP agglomeration pattern has gradually transformed from the original "dual-core" agglomeration pattern to the "multi-core" model. This is closely related to the country’s strong economic base. Additionally, since 2000, the local government has enacted a series of economic reform programs that emphasize the priority development of the agricultural sector while ensuring financial investment in the oil and gas industry and actively developing the manufacturing sector, among others [45].

### Correlation between electricity generation and economic scale

#### High-value regions of electricity generation and economic scale

Suppose the number of high-value countries regarding electricity generation and economic scale in Africa is small and the proportion of those mentioned above is high. In that case, the distribution patterns directly determine the overall pattern of electricity generation and economic scale to a certain extent. The high-value countries are the same, confirming the high correlation between electricity generation and the African economic scale. Based on this, by ranking the electricity generation and GDP of African countries in 2000 and 2019, we found that the electricity generation and economic scale of high-value African countries were mainly concentrated in six countries; South Africa, Egypt, Algeria, Nigeria, Morocco, and Libya. The distribution patterns of these countries did not change significantly over time (Table 1). In 2000, the total electricity generation capacity of the six countries accounted for 80.66% of Africa’s total capacity, whereas the total GDP accounted for 69.08% of Africa’s total GDP. In 2019, these six countries’ total electricity generation capacity and economic scale accounted for 75.49% and 58.98% of Africa’s total electricity generation, although their percentages have decreased. Libya’s GDP ranking declined the most, from 5th place in 2000 to 13th place in 2019. Owing to its geographical location across the sea from Europe, Libya is known as the "northern gate of Africa," with rich and high-quality oil and gas resources, energy exports, and a large economic scale. However, with the outbreak of the civil war in 2011, Libya’s national political rights were split, the security situation deteriorated, and the country was subject to various forms of external intervention. The long-term rivalry between the two governments (the National Salvation Government in Tripoli and the East Government in Tubruga) has led to an inability to form a synergy in energy exploitation and even mutual constraints. This instability has caused Libya’s energy production to plummet, and its energy revenues have also been significantly reduced. The country’s economic scale is difficult, and its growth rate is the lowest across the Africa.

**Table 1:** Electricity generation and economic scale in high-value African countries.

Country	2000				2019			
	Electricity generation		Economic scale (GDP)		Electricity generation		Economic scale (GDP)	
	Rank	Ratio/%	Rank	Ratio/%	Rank	Ratio/%	Rank	Ratio/%
South Africa	1	48.50	1	22.55	1	31.09	2	14.46
Egypt	2	16.19	2	16.61	2	23.01	3	12.44
Algeria	3	5.85	3	9.29	3	9.17	4	7.04
Libya	4	3.57	5	6.48	4	4.52	13	1.68
Morocco	6	3.16	6	6.28	5	4.01	5	4.92
Nigeria	5	3.39	4	7.87	6	3.70	1	18.44
Total	——	80.66	——	69.08	——	75.50	——	58.98

#### Linear correlation between electricity generation and economic scale

**Fig 3:**
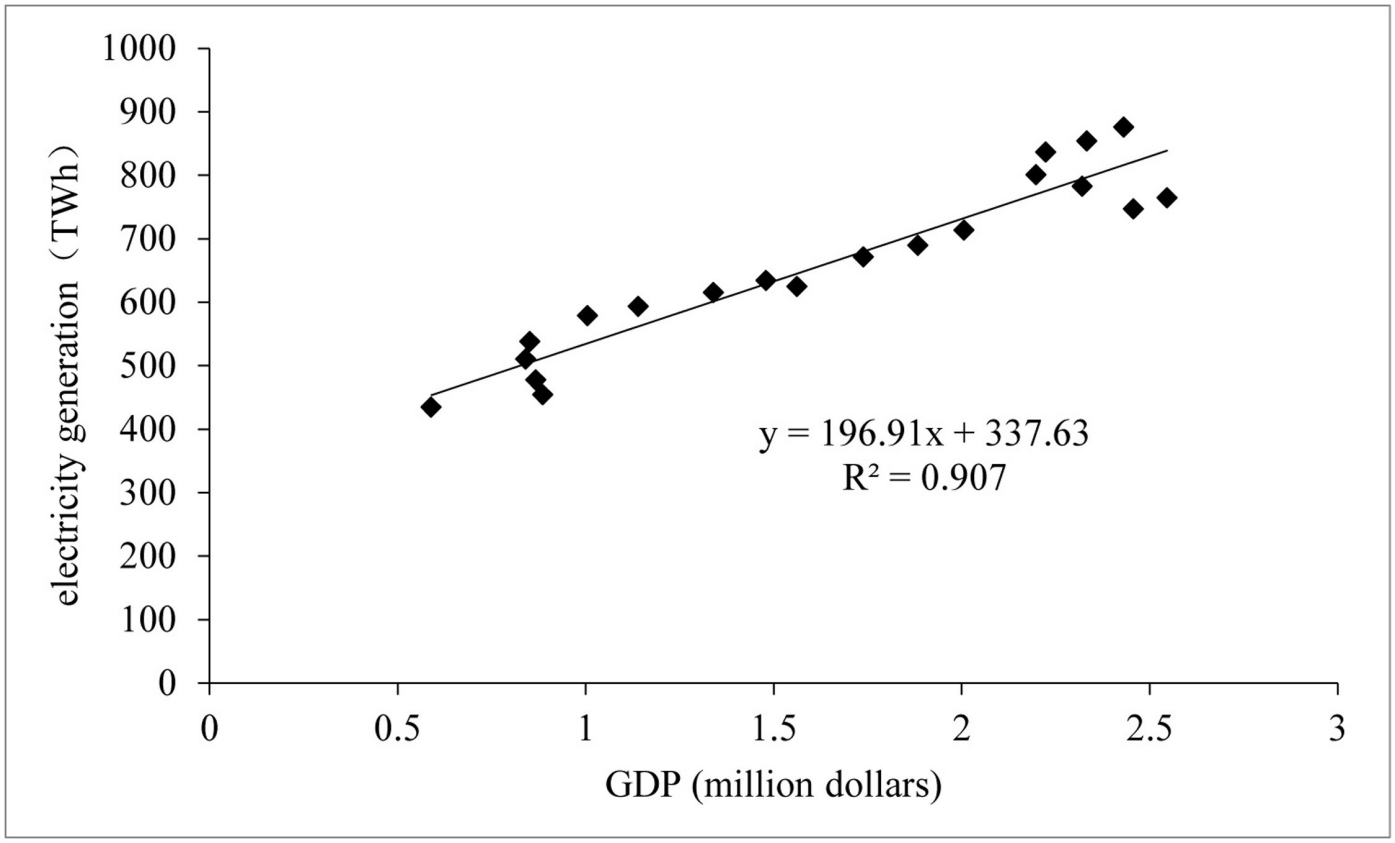
Linear correlation between electricity generation and economic scale in Africa.

Measuring the correlation between electricity generation and the economic scale in Africa revealed a high linear correlation of 0.907 (Fig 3), consistent with the findings of the above analysis. What is the degree of spatial coupling correlation between electricity generation and the African economic scale? To what extent does electricity generation affect the scale of the economy? Based on this, this study attempts to analyze the gravity centers spatial coupling between electricity generation and economic scale in Africa and then adopts the gray correlation analysis method to analyze the gray correlation degree of electricity generation on an economic scale in Africa.

## Spatial coupling of gravity centers of electricity generation and economic scale

### The gravity centers’ trajectories of electricity generation and economic scale

**Fig 4:**
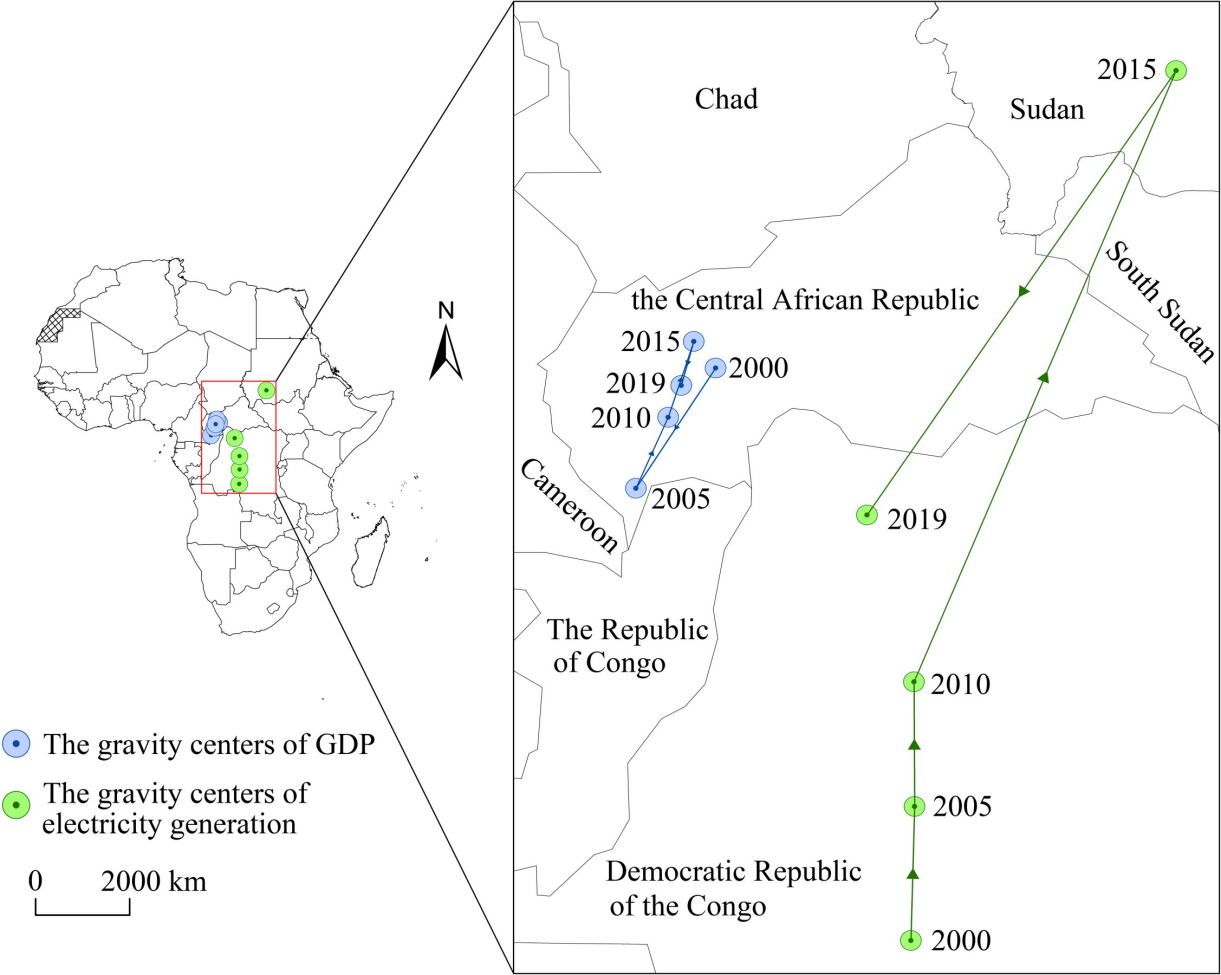
The migration trajectories of gravity centers of GDP and electricity generation.

From a macro perspective, the gravity centers of the economic scale and electricity generation are concentrated in the center of the African continent, including the Central African Republic, Congo (DRC), and Sudan. The gravity centers of economic scale in Africa show a "Southwest-Northeast-Southwest" trajectory (Fig 4), and the five years of gravity centers of economic scale are in the Central African Republic. Specifically, northern countries such as Egypt, Morocco and Libya declined in their share of Africa’s GDP between 2000 and 2005, whereas western countries, notably Nigeria, Ghana, and Gabon, experienced a rapid economic growth. Consequently, the gravity centers of economic scale have shifted to the Southwest, and the economic status of South Africa has declined significantly due to the financial crisis and immigration policy. Its share of Africa’s GDP fell from 23.76% in 2015 to 13.65% in 2019, while the economic status of northern countries began to rise, leading to the transfer of the gravity centers of economic scale to the Northeast. South Africa’s share of Africa’s economic size increased in 2019, while a few countries in West Africa experienced rapid growth in economic size, which boosted a shift in the center of gravity towards the southwest. However, in general, the gravity centers of economic scale move a short distance and their spatial distribution is relatively stable, mainly due to the low level of economic development and the weak foundation of most African countries. The overall relative gap has not changed significantly during the study period, although the size of their economies has increased to some extent.

Compared with the trajectory mentioned above of gravity centers of economic scale, the gravity centers of electricity in African are relatively simple. It presents a "Northeast-Southwest" process, and the distance and speed increase year by year. This is mainly because of the combination of economic development, population growth and urbanization at the onset of the 21st century. Regions relatively more developed, represented by Egypt and Algeria in North Africa, emerged as pioneers in achieving rapid growth in electricity generation, consequently causing a shift in the gravitational centers towards the north. Since 2010, East Africa has witnessed substantial improvements in the investment climate and regional connectivity, resulting in a rapid increase in the number of people gaining access to electricity. This directly contributed to a shift in the gravitational centers of electricity generation toward the Northeast. After 2015, with the advancement of initiatives such as the "Belt and Road" and African integration, African nations embarked on extensive privatization reforms in the electricity sector. This attracted significant foreign investment toward local power infrastructure development, notably from Chinese investors. Notably, there was significant growth in electricity generation in the West and Central African regions, represented by Nigeria and Angola. Simultaneously, the increasing demands for electricity generation stemming from economic recovery further fueled this shift, ultimately leading to a reorientation of the power generation center towards the south-west by 2019.

The shift in the trajectory of the gravity centers of economic scale and electricity generation in the north-south direction has been notably more significant than in the east-west direction. This discrepancy arises because while Eastern and Western Africa experienced relatively modest disparities in electricity generation, their differences are not so pronounced. In contrast, Sub-Saharan Africa and South Africa continue to serve as pivotal hubs for electricity generation and economic scale in on the continent. Consequently, movements in their respective indicators consistently dominate the trajectory shift of the gravity centers.

### Spatial coupling of gravity centers of electricity generation and economic scale

**Fig 5:**
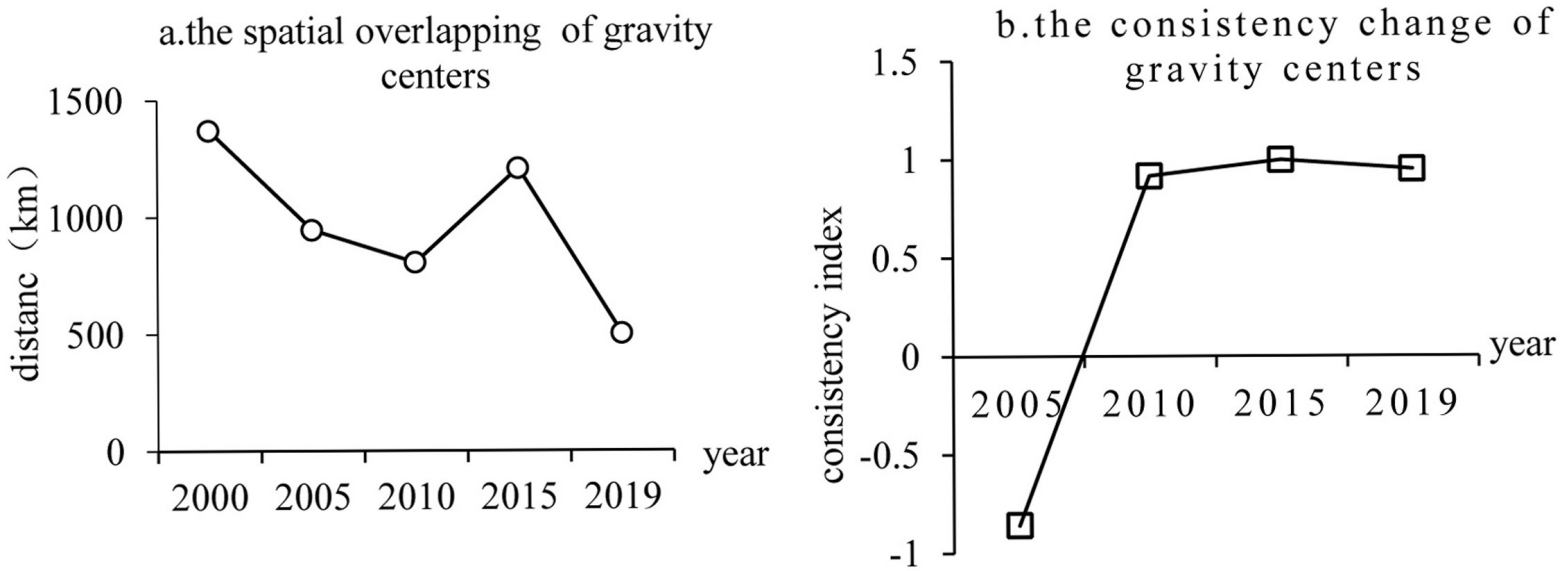
The changes of the spatial overlapping and consistency index between GDP gravity centers and electricity generation gravity centers in Africa.

In analyzing the spatial overlap of the gravity centers, it was observed that the distance between the gravity centers of electricity generation and the gravity centers of economic scale in Africa ranged from 501 km to 1,371 km. Furthermore, it showed a general decreasing trend (Fig 5). Notably, a sharp decline in 2015 indicates the growing spatial consistency between these centers, a trend that has intensified in recent years. Regarding directional consistency, the index between the gravity centers of electricity generation and economic scale in Africa steadily increased from -0.86 in 2005 to 0.95 in 2019, stabilizing after that near to 1.The spatial coupling process between the electricity generation and the gravity centers of economic scale exhibited a rapid shift from reversal to uniformity, indicating a high degree of consistency between the gravity centers of electricity generation and the gravity centers of economic scale in Africa. While in 2005, the transfer speed of electricity generation lagged behind that of the gravity centers of economic scale. Still, at the following time points, the gravity centers of electricity generation and the gravity centers of economic scale remained relatively stable and had on obvious codirectional transfer process. Based on this, African nations should prioritize improving power infrastructure to promote sustained electricity generation growth. This should align with, regional economic development needs while, creating a favorable investment environment and establishing cross-regional power collaboration mechanisms. These efforts aim to reduce operating costs and significantly expand power coverage, thus supporting Africa’s sustained and healthy economic development.

### Gray correlation analysis between electricity generation and economic scale

#### Selection of correlation factors

Based on the above analysis and verification that there is a strong spatial coupling relationship between the gravity centers of electricity generation and the gravity centers of economic scale in Africa, combined with the basic data of electricity generation and economic scale of African countries from 2000 to 2019, this study uses the gray correlation analysis method to measure the correlation between Africa’s electricity generation and economic scale. Considering the main influencing factors related to the scale of the economy, but due to the difficulties in obtaining data in some countries, the final selection of six factors: foreign direct investment, openness to the other parts worldwide, infrastructure, labor force, urbanization level, and electricity generation involved 11 independent variables (Table 2). Following a literature review and the analysis of the spatial coupling of gravity centers, this study hypothesizes that electricity generation in Africa can significantly promote the expansion of the economic scale. In addition, ten control variables significantly impact the scale of the economy.

**Table 2:** The factors affecting location choice of economic scale in Africa.

Impact factors	Representative indicators	References
Electricity generation	The amount of electricity produced by each country (GWh)	Obser’ Eve [1] Keho Y [2] Balachandra P [3]
Foreign Investment	FDI (100 million USD)	Wenhao W [46] Puman O [47]
Openness to the outside world	Total imports and exports as a percentage of GDP (%)	Jayanthakumaran K [48]
Infrastructure	Number of mobile phones per 100 people (set)	Irem B [49]
	Number of fixed telephones per 100 persons (set)	
	Correlation coefficient of liner shipping	
	Air transport cargo volume (10000 tons)	
	Passenger traffic by air (10000 persons)	
	Total kilometers of railway (10000 km)	
The labor force	The quantity of labor force (10000 persons)	Liyuan S [50] Douglas G [51]
Level of urbanization	The proportion of urban population to the total population (%)	Tao H [52]

Foreign Direct Investment (FDI), mainly by multinational companies, has become an important force in promoting the sustained and rapid development of regional economies [46]. Extensive empirical studies using national and corporate data confirm that FDI contributes to economic growth in recipient countries by providing physical capital, advanced technology, and management expertise [47]. Moreover, the direct effect of FDI on regional economic development is increased regional capital, which is directly related to changes in the regional economic scale. Therefore, FDI was chosen as a foreign direct investment measure. Greater openness to the outside world, such as through the establishment of export processing zones and free trade area zones, will help attract FDI by giving foreign investors preferential policies, facilities and services [48]. Consequently, the economic scale will further increase the economic scale, as measured by the specific choice of gross imports and exports as a proportion of GDP. Sound infrastructure is essential in facilitating economic development, especially in developing countries. It serves as a crucial external condition for achieving significant scale in economic growth, reducing trade and transport costs. And according to neoclassical growth theory, infrastructure investment ultimately promotes economic growth [49], which can be described by a fixed telephone number per 100 people, mobile phone number per 100 people, liner transport coefficient, the amount of freight transported by air, number of passengers transported by air, and the total kilometers travelled by rail. Labor supply is an important source of economic growth, and its changes will inevitably affect economic growth. Many scholars have long recognized that China’s abundant labor resources important in promoting long-term economic growth [50]. In less developed regions (such as Africa), the quantity of labor tends to be more attractive for investment in labor-intensive firms, as measured by the specific choice of labor quantity indicator. An urban industrial workforce can accelerate industrialization and significantly affect a country’s economy [51]. In many emerging economies, the relationship between urbanization and economic growth is extremely strong, and the positive impact of urbanization on economic growth has been widely accepted by national and local governments [52], specifically by choosing the urban population as a proportion of the total population for measurement.

### The analysis of gray correlation degree

This study examines the correlation between electricity generation and the economic scale of African countries from 2000 to 2019, using a gray correlation degree and a DPS data processing system for initial value analysis. Results indicate that out of the 11 representative indicators, 10 correlate above 0.65 with the economic scale (Table 3), highlighting their significant impact on the economy’s size. Electricity generation is the highest gray correlation degree with GDP, reaching 0.7590, signifying a strong correlation with the economic scale. This reaffirms the notion of electricity production data serving as a "Barometer" of economic performance and growth in Africa, where electricity demand rises alongside economic expansion. Additionally, the air transport passenger volume, FDI, labor force quantity, shift transportation correlation coefficient, and economic scale’s gray correlation degree also show notable correlations. Air and ship transport have been the two most rapidly emerging modes of transport in recent years [48], matching the trend of rapid economic development in Africa and greatly strengthening the economic and trade exchanges between African countries and other regions; Foreign investment in Africa directly affects the changes in the scale of the African economy, which is due to the shortage of endogenous development factors such as capital, talents and technology within the African region, which directly leads to an increase in the total volume of the regional economy. Simultaneously, the spatial aggregation of production factors promotes the self-strengthening process of the labor force and capital flow, and the level of human capital directly affects the scale of regional economic changes, especially in the labor shortage in Africa. The total length of railways, proportion of total imports and exports in GDP (%), air transport cargo volume, and proportion of urban population in the total population (%) are less gray-correlated with the economic scale. This is because the overall development of rail transport in Africa is slow, and the spatial distribution is extremely uneven, with railway powers such as South Africa and rail-zero countries such as Niger, which is not consistent with the development trend at an economic scale [48]. The level of opening-up [49] urbanization and air cargo transport promote economic growth, but the economic spillover effect is still limited. In contrast, the number of fixed phones per 100 people and the number of mobile phones per 100 people had the smallest gray correlations with the economic scale. This is mainly because of the lateness of Africa’s telecommunications industry, which is still in its initial stage, and the development of mobile communications throughout Africa is characterized by a “weak foundation, rapid momentum and regional imbalance.” Under these conditions, the impact of communications infrastructure on the economic scale is minor. Based on the above analysis, the gray correlation analysis results are consistent with the center of gravity spatial coupling pattern results.

**Table 3:** Grey correlation degree between economic scale and each factor in Africa from 2000 to 2019.

Impact factors	Gray correlation degree	Ranking
Electricity generation	0.7590	1
Passenger traffic by air	0.7577	2
FDI	0.7428	3
The quantity of labor force	0.7263	4
Correlation coefficient of liner shipping	0.7034	5
Total kilometers of railway	0.6990	6
Total imports and exports as a percentage of GDP (%)	0.6961	7
Air transport cargo volume	0.6855	8
The proportion of urban population to the total population (%)	0.6558	9
Number of fixed telephones per 100 people	0.6554	10
Number of mobile phones per 100 people	0.3476	11

**Fig 6:**
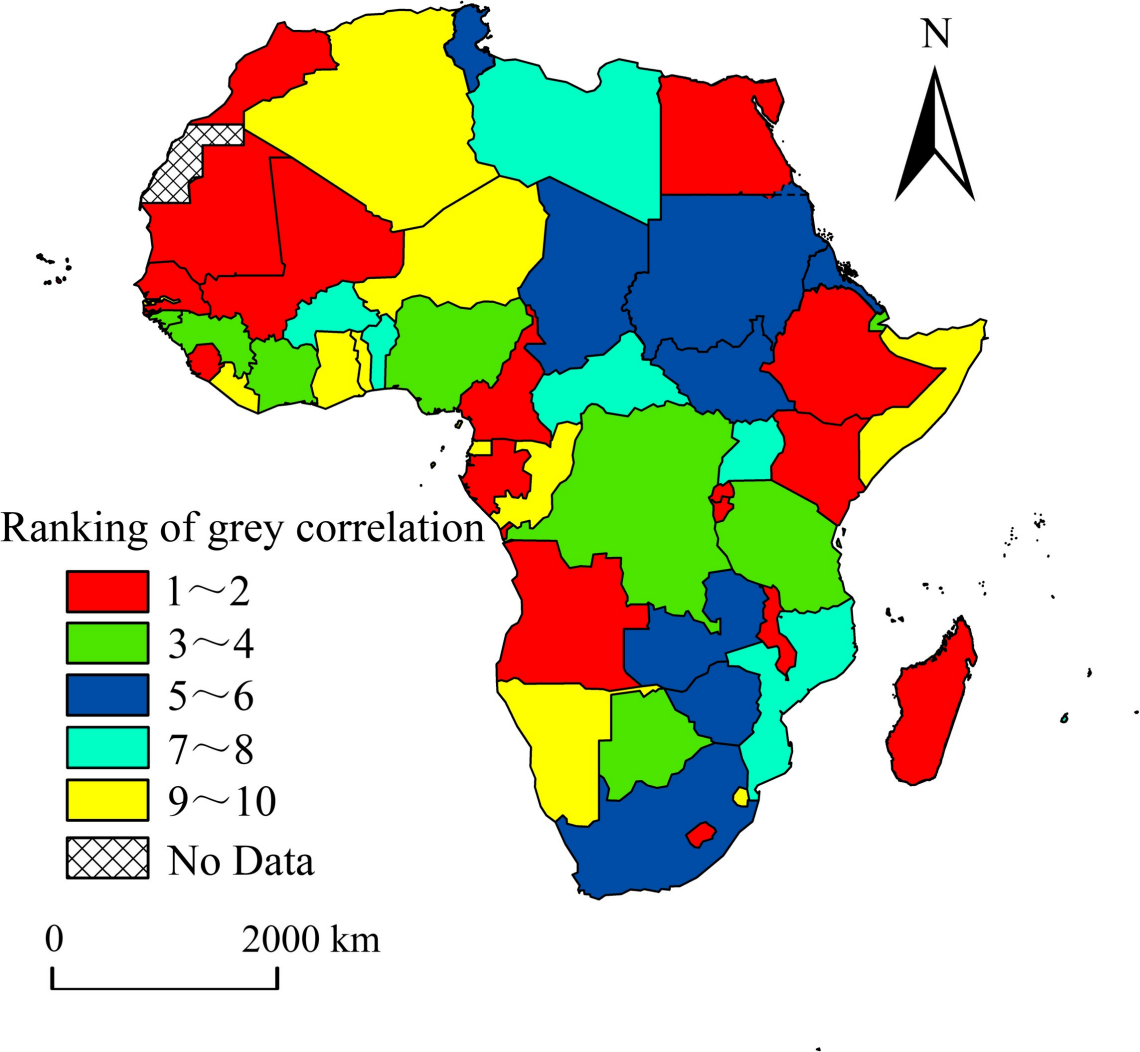
The spatial pattern of ranking for grey correlation degree between electricity generation and economic scale in Africa.

Based on the above analysis, the spatial patterns of the gray correlation degree ranking of electricity generation and economic scale of African countries from 2000 to 2019 were further analyzed and compared using the gray correlation analysis method (Fig 6). The 18 countries with the highest gray correlations between electricity generation and economic scale are North African countries (Egypt, Morocco, and Mauritania), four West African countries (Cape Verde, Mali, Senegal, and Sierra Leone) four East African countries (Burundi, Ethiopia, Kenya, and Seychelles), there are three Central African countries (Cameroon, Gabon and Rwanda) and four South African countries (Angola, Lesotho, Madagascar and Malawi). Sixteen countries are ranked in the order of 3–4 and 5–6, distributed across North Africa, West Africa, Central Africa, East Africa and South Africa. In summary, the number of countries with a gray correlation between electricity generation and economic scale in the upper-middle level accounts for nearly two-thirds of Africa’s total, with a large number and wide distribution range. This further reflects that the important impact of electricity generation on the economic scale is universal across African countries. Therefore, while actively promoting economic development in African countries, local governments should increase investments in fixed electricity assets and attract foreign enterprises to invest in electricity through unified and stable market policies to bridge the financing gap. Simultaneously, the strengthening of the integration process in Africa, the removal of barriers to electricity trade, the promotion of electricity trade, and the reduction of the cost of electricity use will contribute to the steady growth of the economic scale.

## Conclusion and discussion

### Conclusion and policy recommendation

Using the spatial interpolation method in ArcGIS and the gray correlation analysis method in DPS, combined with the Africa Statistical Yearbook 2001–2020, the spatial pattern of electricity generation and economic scale in Africa since the beginning of the 21st century and the spatial correlation between electricity generation and economic scale were analyzed. The results reveal the following. (1) The spatial difference in electricity generation is significant, mainly concentrated in Northern and Southern Africa, and this spatial pattern dose not changed significantly with time, which is similar to the spatial pattern of the economic scales. (2) Africa’s electricity generation and economic scale are concentrated in South Africa, Egypt, Algeria, Nigeria, Morocco, and Libya, and the distribution patterns of these high-value countries have not changed significantly over time. This was further confirmed via a correlation analysis, where the linear correlation coefficient between the two was as high as 0.907. (3) Both the economic scale and the gravity centers of electricity generation are concentrated in the middle of the African continent, and the distance moved from north to south is much greater than the distance moved from east to west. The gravity centers of the African economic scale present a "Southwest-Northeast-Southwest" trajectory, and the gravity centers of electricity generation present a "Northeast-Southwest" trajectory. From the perspective of spatial overlap of gravity centers, the distance between the gravity centers of economic scale in Africa and the gravity centers of electricity generation generally shows a downward trend, and the consistency of the spatial distribution between the two continues to increase. (4) Electricity generation in Africa has the highest gray correlation degree with the economic scale; that is, the amount of electricity generation greatly affects the economic scale; which is consistent with the result of the Institute of Water Resources and Electric Power Information of the Ministry of Energy of China [18]. In addition, the number of African countries with a gray correlation degree of medium to upper level with economic scale accounts for nearly 2/3 of the proportion in Africa, with a large number and wide distribution range.

As there are obvious spatial differences in the electricity generation and economic scale of different countries, and the profound impact of African power generation on the economic scale, this study puts forward corresponding countermeasures and suggestions based on the reality of the African continent.

1. African governments should make full use of their local comparative advantages, actively cooperate with other countries in the world, improve foreign aid and trade mechanisms, attract foreign investment, strive to break the negative effect of the "resource curse," accelerate the development of resources and the transformation of industrial structure, and provide strong economic support for the steady increase in electricity generation. Simultaneously, African countries should improve the intervention capacity of African governments, establish special financial funds, accelerate the construction of electricity facilities, and extend the service life of electricity infrastructure by introducing new equipment and technologies to improve the sustainability of electricity generation in Africa. Furthermore, it is necessary to accelerate the interconnection of electricity grids, build a cross-border intercontinental electricity grid interconnection route, and resolve the unbalanced distribution of electricity resources and consumption regions.
2. For electricity enterprises, considering the development of fossil energy, hydropower, wind power, photovoltaic electricity generation, and biomass electricity generation systems built in resource-rich areas, and efforts made to control electricity generation costs and expand electricity generation channels. Electricity enterprises should also develop and support each other in various modern industries to meet productive energy demands, cultivate the willingness and ability of power consumers to pay, and alleviate the dilemma of the power consumption market. Improve the power production structure planning, foster the development of the modern power enterprise systems, and achieve stable and controllable power production and operation.

## Discussion

Focusing on Africa as the research domain and electricity generation coupled with economic scale as the research objects, this study delves into the spatial pattern and barycentric coupling degree of electricity generation and economic scale across the continent. By adopting the gray correlation analysis method, this study analyzes the degree of correlation between electricity generation and economic scale in Africa, shedding light on their spatial correlation since the turn of the 21st century. Moreover, the study puts forth various scientifically policy recommendations tailored to diverse governmental departments across African nations. These suggestions serve as valuable references for fostering the coordinated development of electricity generation and economic scale throughout the continent. However, this study only incorporates representative and accessible data indicators for evaluation due to challenges in accessing statistical data from African countries and the multifaceted nature of factors influencing the continent’s economic scale. Moreover, correlation techniques cannot discern the direction of impact; they merely indicate the degree of the relationship between the variables. These limitations underscore the need for further investigation and research in this domain.

## Supporting information

S1 File (PDF)

S2 File (XLSX)
