# Supplementary material for: Spatial correlation between electricity generation and economic scale in Africa
Source: PLoS One. 2024 Jun 24;19(6):e0300627. doi: 10.1371/journal.pone.0300627 (PMC11195941; doi:10.1371/journal.pone.0300627)
Supplement: S1 File — (PDF) [file pone.0300627.s001.pdf]

Supporting information

Table S1 The electricity generation of Africa countries in 2000 and 2019, presented in Fig 1.

Country	2000/GWh	2019/GWh
Republic of Cape Verde	146	515
Republic of Cote d'Ivoire	4813	10474
Republic of Ghana	7224	15380
Republic of Liberia	310	130
Kingdom of Morocco	13715	35131
Western Sahara	0	0
Burkina Faso	390	1137
Republic of Guinea	768	2029
Republic of Guinea-Bissau	58	193
Republic of Mali	412	2893
Islamic Republic of Mauritania	234	859
Republic of Senegal	1671	5031
Republic of Sierra Leone	95	452
Republic of The Gambia	132	343
Republic of Djibouti	180	31
State of Eritrea	209	462
Federal Democratic Republic of Ethiopia	1696	14034
Republic of the Sudan	2450	17328
Republic of Uganda	1582	4318
People's Democratic Republic of Algeria	25412	80235
Great Socialist People's Libyan Arab Jam	15496	39525
Tunisian Republic	10095	20694
Republic of Benin	84	229
Republic of Cameroon	3480	8573
Central African Republic	107	154
Republic of Chad	92	309
Republic of Equatorial Guinea	23	480
Republic of Niger	190	559
Federal Republic of Nigeria	14727	32380
Sao Tome and Principe	18	100
Togolese Republic	275	521
Arab Republic of Egypt	70337	201415
Somalia	250	432
Republic of Botswana	1114	3102
Republic of Burundi	101	266
Republic of Kenya	4323	11995
Rwandese Republic	113	562
United Republic of Tanzania	2479	8067
Republic of Zambia	7798	14560

Republic of Zimbabwe	6995	8073
Union of the Comoros	19	77
Republic of Malawi	1226	2871
Republic of Mozambique	8849	17439
Republic of Angola	1445	13507
Republic of the Congo	298	2745
Democratic Republic of the Congo	6018	11402
Gabonese Republic	1315	2787
Kingdom of Lesotho	290	540
Republic of Namibia	1414	1571
Republic of South Africa	210670	272102
Kingdom of Swaziland	470	860
Republic of Madagascar	780	2636
Republic of Mauritius	1777	3319
Republic of Seychelles	188	499

Table S2 The GDP of Africa Countries in 2000 and 2019, presented in Fig 2.

**Country**	**2000gdp/ million dollars**	**2019gdp/ million dollars**
Republic of Cape Verde	571	1982
Republic of Cote d'Ivoire	10420	58539
Republic of Ghana	4980	66998
Republic of Liberia	661	3176
Kingdom of Morocco	37060	119701
Western Sahara	0	0
Burkina Faso	2618	15991
Republic of Guinea	2995	12340
Republic of Guinea-Bissau	216	1345
Republic of Mali	2640	17419
Islamic Republic of Mauritania	1081	7600
Republic of Senegal	4681	23579
Republic of Sierra Leone	636	4119
Republic of The Gambia	421	1818
Republic of Djibouti	556	3346
State of Eritrea	706	1982
Federal Democratic Republic of Ethiopia	7970	92796
Republic of the Sudan	12367	36535
Republic of Uganda	6099	34753
People's Democratic Republic of Algeria	54796	171162
Great Socialist People's Libyan Arab Jam	38214	40951
Tunisian Republic	19444	38796
Republic of Benin	2360	14392

Republic of Cameroon	9290	38863
Central African Republic	960	2117
Republic of Chad	1386	10523
Republic of Equatorial Guinea	1178	11025
Republic of Niger	1667	12928
Federal Republic of Nigeria	46386	448120
Sao Tome and Principe	77	476
Togolese Republic	1291	5460
Arab Republic of Egypt	97954	302346
Somalia	1219	4942
Republic of Botswana	5633	18474
Republic of Burundi	709	3454
Republic of Kenya	12604	95410
Rwandese Republic	1735	10354
United Republic of Tanzania	10186	60810
Republic of Zambia	3238	23310
Republic of Zimbabwe	5714	21818
Union of the Comoros	202	1166
Republic of Malawi	1744	8128
Republic of Mozambique	4183	15195
Republic of Angola	9917	86158
Republic of the Congo	3221	12911
Democratic Republic of the Congo	4335	49816
Gabonese Republic	5069	16875
Kingdom of Lesotho	783	2380
Republic of Namibia	3909	12364
Republic of South Africa	132964	351354
Kingdom of Swaziland	1490	4656
Republic of Madagascar	3866	14105
Republic of Mauritius	4583	14048
Republic of Seychelles	738	1702

Table S3 The electricity generation and economic scale in Africa in the period of 2000-2019, presented in Fig 3.

Year	GDP/million dollars	Electricity generation / Twh
2000	0.589723	434.353
2001	0.886788	454.235
2002	0.867751	477.569
2003	0.841441	510.366
2004	0.851858	537.894
2005	1.005555	578.663
2006	1.140594	592.83

2007	1.339944	614.788
2008	1.56246	624.705
2009	1.479652	633.546
2010	1.738634	670.774
2011	1.883468	689.224
2012	2.006779	713.493
2013	2.456304	747.007
2014	2.545851	764.058
2015	2.320601	782.531
2016	2.198115	800.875
2017	2.223807	836.353
2018	2.333043	853.594
2019	2.43061	875.322

Table S4 The gravity centers of GDP (XE, YE,) and the gravity centers of electricity generation (XP, YP) in 2000,2005,2010,2015 and 2019, presented in Fig 4.

**Year**	X_E	Y_E	X_P	Y_P
2000	17.92159706	5.962506351	21.74965291	-5.820933588
2005	16.35811661	3.487276039	21.82121998	-3.063753275
2010	16.99126133	4.948553668	21.81041024	-0.497916668
2015	17.49220507	6.508428762	26.9498926	12.08441268
2019	17.24708559	5.603127779	20.89009967	2.935768834

Table S5 The distance between the gravity centers of economic and electricity generation in 2000,2005,2010,2015 and 2019, presented in Fig 5.

**Year**	X_E-X_P	Y_E-Y_P	$(x_E - x_p)^2$	$(y_E - y_p)^2$	$\sqrt{(x_E - x_p)^2 + (y_E - y_p)^2}$ （decimal degree）	$\sqrt{(x_E - x_p)^2 + (y_E - y_p)^2}$ (kilometers)
2000	-3.828055854	11.78343994	14.65401162	138.8494568	12.38965167	1370.672424
2005	-5.463103364	6.551029315	29.84549836	42.91598508	8.5300342	945.606737
2010	-4.819148911	5.446470336	23.22419623	29.66403912	7.27242981	806.136796
2015	-9.457687522	-5.57598392	89.44785327	31.09159668	10.97904595	1207.921626
2019	-3.643014085	2.667358946	13.27155162	7.114803745	4.515125178	500.525949

Table S6 The consistency index cosθ between economic gravity centers and electricity generation gravity centers in Africa, presented in Fig 5.

**Year**	$\Delta x_E{}^2 + \Delta y_E{}^2$	$\Delta x_p{}^2 + \Delta y_p{}^2$	$\sqrt{\left(\Delta x_E{}^2 + \Delta y_E{}^2\right)\left(\Delta x_p{}^2 + \Delta y_p{}^2\right)}$	$\Delta x_E \Delta x_p + \Delta y_E \Delta y_p$	cosθ
2005	8.571236206	7.607165121	8.07482564	-6.936549987	-0.859034027
2010	2.536204544	6.583634347	4.086250524	3.742555508	0.915889881
2015	2.684154941	184.7292907	22.26751083	22.2014537	0.997033475
2019	0.879653431	120.4187745	10.29207405	9.767649592	0.949045794

Table S7 The value of grey correlation degree between electricity generation and economic scale in Africa, presented in Fig 6.

Country	Electricity generation	FDI	Total imports and exports as a percentage of GDP (%)	Number of mobile phones per 100 people	Number of fixed telephones per 100 people	Correlation coefficient of liner shipping	Air transport cargo volume	Passenger traffic by air	Total kilometers of railway	The quantity of labor force	The proportion of urban population to the total population
Angola	0.9882	0.914	0.911	0.89	0.8836	0.8808	0.8712	0.8644	0.86	0.8508	0.339
Burundi	1.028	1.0228	0.9847	0.9747	0.9578	0.9549	0.9346	0.9247	0.9174	0.8189	0.4661
Benin	1.0426	1.0276	1.0259	0.9943	0.9889	0.9855	0.9786	0.9566	0.937	0.7813	0.5362
Burkina faso	1.0368	1.0177	1.0166	1.0154	1.0142	1.0076	0.9804	0.9694	0.9583	0.9232	0.4507
Botswana	1.0495	1.035	1.0118	0.9979	0.9881	0.9873	0.974	0.9724	0.9673	0.9384	0.7532
Central African Republic	1.0343	1.0177	1.0169	1.01	1.0064	1.0056	1.0017	0.9898	0.974	0.7097	0.3945
Cote d'Ivoire	0.9215	0.843	0.8418	0.8373	0.8059	0.7977	0.7823	0.7805	0.7397	0.7372	
Cameroon	1.0204	0.9909	0.9821	0.9721	0.9652	0.9557	0.939	0.9144	0.9135	0.8974	0.3594
Democratic Republic of the Congo	1.0855	1.0846	1.0685	1.0631	1.0584	1.0503	1.0297	1.0162	1.0123	1.0037	0.4191
Congo	1.0755	1.0447	1.044	1.0431	1.0424	1.0406	1.014	1.0131	0.9863	0.8728	0.5729

Comoros	1.084	1.06	1.035	1.034	1.026	1.024	1.009	1.009	1.009	1.009	0.4408
Cabo Verde	0.9238	0.9226	0.9053	0.8372	0.7587	0.7428	0.7405	0.6979	0.6934	0.5527	0.4241
Djibouti	1.0728	1.0725	1.0343	1.0336	1.0318	1.031	1.0218	1.0135	0.9794	0.8173	0.7129
Algeria	1.0633	1.0574	1.0551	1.0458	1.0409	1.0354	1.0177	1.0114	1.0079	0.9891	0.3126
Egypt	0.9893	0.9393	0.9054	0.8978	0.8971	0.888	0.871	0.8545	0.8444	0.719	
Eritrea	1.094	1.08	1.08	1.064	1.049	1.043	1.043	1.03	1.03	1.001	0.601
Ethiopia	1.0741	1.0635	1.0564	1.0433	1.0382	1.0337	1.0336	1.0136	1.0127	1.0023	0.6785
Gabon	0.6624	0.6346	0.6289	0.6206	0.5965	0.5858	0.5836	0.5638	0.5444	0.5223	0.3836
Ghana	0.9128	0.8938	0.8623	0.862	0.8559	0.8394	0.8361	0.8276	0.8205	0.8167	0.3963
Guinea	1.0239	1.0075	0.9997	0.9985	0.9905	0.9826	0.9768	0.958	0.949	0.92	0.4735
Gambia	1.0479	1.0454	1.0348	1.0332	1.0022	0.9962	0.9906	0.987	0.986	0.8793	0.5223
Guinea-Bissau	1.089	1.089	1.089	1.084	1.054	1.054	1.032	1.03	1.025	1.02	0.392
Equatorial Guinea	0.651	0.5388	0.5269	0.5146	0.503	0.49	0.486	0.4587	0.4328	0.4309	0.3689
Kenya	1.0013	1.0006	0.9838	0.9814	0.9508	0.9494	0.9487	0.9435	0.9382	0.9133	0.37
Liberia	1.0891	1.0533	1.0522	1.0492	1.0446	1.0135	1.0123	1.0112	0.9874	0.926	0.6006
Libya	1.039	1.0189	1.0136	1.0094	1.0041	1.0004	0.9985	0.9901	0.9634	0.8762	0.5379
Lesotho	0.9847	0.955	0.9238	0.9204	0.9078	0.9044	0.9023	0.9007	0.8258	0.8225	0.5495
Morocco	0.7854	0.7522	0.7272	0.6669	0.6566	0.6266	0.6262	0.6248	0.5325	0.374	0.3421
Madagascar	1.0165	1.0013	0.9963	0.9755	0.9753	0.9541	0.9489	0.9429	0.9326	0.9122	0.5584
Mali	1.0426	1.025	1.0243	1.023	1.0191	1.0156	0.9996	0.997	0.9961	0.9924	0.5866
Mozambique	0.9433	0.9323	0.9216	0.8856	0.8833	0.8807	0.8792	0.879	0.864	0.7954	0.4468
Mauritania	1.072	1.072	1.066	1.066	1.052	1.05	1.042	1.039	1.039	1.028	0.2905
Mauritius	0.9963	0.7868	0.7281	0.7219	0.7136	0.7008	0.692	0.6798	0.6753	0.457	0.4556
Malawi	0.9398	0.9181	0.9072	0.8995	0.8717	0.856	0.8486	0.8394	0.821	0.7872	0.4303

Namibia	0.9196	0.8551	0.7355	0.7158	0.7089	0.7052	0.6966	0.6753	0.6653	0.6369	0.3491
Niger	1.0789	1.076	1.0686	1.0678	1.05	1.0471	1.0342	0.9959	0.9854	0.9713	0.6126
Nigeria	1.0739	1.0727	1.0572	1.0562	1.0556	1.0364	1.0096	1.0086	1.0076	0.9964	0.5247
Rwanda	1.0221	0.974	0.9617	0.9605	0.9252	0.9237	0.9188	0.9026	0.8923	0.6698	0.4744
Sudan	1.0562	1.0476	1.0465	1.0181	1.018	0.9936	0.9921	0.9887	0.9816	0.9799	0.4221
Senegal	0.8528	0.8464	0.8441	0.8411	0.832	0.8208	0.8051	0.7861	0.7733	0.7581	0.3663
sierra leone	1.0291	1.0246	1.0051	0.9916	0.9766	0.9709	0.9706	0.9684	0.9612	0.9509	0.4676
Somalia	1.0718	1.0636	1.0573	1.0543	1.0303	1.0055	0.998	0.9975	0.9925	0.832	0.4652
Sao Tome and Principe	1.095	1.095	1.091	1.091	1.091	1.084	1.084	1.041	1.041	1.018	0.4136
Eswatini	0.7785	0.773	0.7705	0.7543	0.7413	0.7394	0.7267	0.6968	0.666	0.5732	0.4443
Seychelles	0.7635	0.7291	0.6682	0.6547	0.6026	0.5598	0.4984	0.4606	0.402	0.3516	0.2244
Chad	1.0339	1.0317	1.0197	1.0149	0.9974	0.9782	0.9626	0.9456	0.945	0.9317	0.4414
Togo	1.0291	0.9483	0.9358	0.9317	0.912	0.9103	0.903	0.894	0.8827	0.8696	0.4037
Tunisia	1.0697	1.0689	1.0642	1.0623	1.053	1.0477	1.0419	1.024	1.0232	1.0133	0.3307
United Republic of Tanzania	1.0544	1.0481	1.0476	1.0275	0.9946	0.9928	0.9846	0.9709	0.9657	0.9651	0.382
Uganda	0.9492	0.916	0.9042	0.904	0.8952	0.8951	0.8892	0.8515	0.8074	0.7475	0.4827
South Africa	0.8342	0.7449	0.6107	0.5408	0.5359	0.5275	0.5196	0.4963	0.4718	0.4617	0.3587
Zambia	0.8984	0.8984	0.8946	0.8936	0.8785	0.8645	0.8554	0.8506	0.8483	0.5644	0.5063
Zimbabwe	0.8981	0.882	0.851	0.842	0.8417	0.8362	0.7751	0.7414	0.7375	0.5615	0.4138
